# Editorial: Training & treatment in child mental health services (CAMHS): Novel and collaborative approaches

**DOI:** 10.3389/fpsyt.2022.997647

**Published:** 2022-08-09

**Authors:** Alexis Revet, Cheryl A. Kennedy

**Affiliations:** ^1^Department of Child and Adolescent Psychiatry, Toulouse University Hospital, Toulouse, France; ^2^CERPOP/UMR1295, University of Toulouse, Inserm, UPS, Toulouse, France; ^3^Department of Psychiatry, Rutgers New Jersey Medical School, Newark, NJ, United States; ^4^Department of Psychiatry, University Hospital, Newark, NJ, United States

**Keywords:** vulnerable children, mental illness in children, training for CAMHS, effective treatments in CAMHS, interprofessional treatment for CAMHS

Child and Adolescent Psychiatry and mental health services in the twenty-first century are facing major challenges that require multiple competences and a strong trans-disciplinary and interprofessional input. Epidemiological data now clearly show that mental health care for children and adolescents should be a public health priority in most countries of the world (1, 2). Globally, about half of all mental disorders occur before the age of 14 and three quarters before the age of 25 (1, 3). In addition, 25% of disability-adjusted life years for mental and substance use disorders occur in young people (4). Too often neglected or minimized, the psychosocial risk factors that have a negative impact on the development of children and adolescents are multiple, including psycho-traumatic factors (migration, wars, terrorism, natural disasters, pandemics, domestic, and community violence) as well as risks linked to socio-economic precariousness (poverty, lack of education, child labor, sexual discrimination) (5). At the global level, the very low number of child and adolescent psychiatrists is of particular concern, and is even more pronounced in low-income countries. More worryingly, a downward trend in recruitment to child and adolescent psychiatry is observed in many countries (6).

Given the complexity of these issues and the multiplicity of challenges, the objectives of this Research Topic were broad, potentially integrating contributions of scientific knowledge from various fields (technology, epidemiology, genomics, protective factors, psychotherapeutic approaches, biological treatments, and others) in order to better understand the problems of children and adolescents with psychiatric disorders, and to advance treatment modalities and innovative approaches, in a biopsychosocial, multicultural, inter- and intra-professional and multimodal perspective.

A series of four surveys were conducted to explore the experience of new and collaborative approaches to training and treatment in children's mental health services, summarized below.

You et al. retrospectively studied the effectiveness of acupuncture treatment of tic disorder in children over a 12-week period. This work showed that, compared with the control group, the reduction in the Yale Global Tic Severity Scale (YGTSS) total score after 12 weeks of treatment was greater for the acupuncture group (OR = 2.94, 95% CI: 1.03–8.39, *p* = 0.04), and this association was stronger for subjects who had significant vocal tics (β = 0.29, 95% CI: 0.88–2.68, *p* = 0.001). The authors of this work have thus provided preliminary evidence supporting the therapeutic effect of acupuncture for tic disorder in children, which could therefore be an effective and well-supported adjuvant treatment in children with this indication, particularly for vocal tics.

Kilicel et al. focused on the transition in psychiatry, that refers to the period when young people move from child and adolescent mental health services to adult mental health services. This period is marked by a strong discontinuity with a potential impact on the young adult's disorders and subsequent follow-up, in both psychiatric (7) and non-psychiatric disorders (8). The objective of this study was to describe the architecture of public mental health providers in Switzerland and to compare it to EU countries, around this issue of transition in psychiatry. Interestingly, the results of this work have shown that, although Switzerland has some of the highest socio-economic resources available for mental health care services in Europe, Swiss cantons seem to experience comparable difficulties and have similar demands and needs regarding transition issues that other countries have. These results are important in that they show that the level of resources invested in mental health care may not be the most important parameter to ensure a quality transition. Indeed, as the authors point out, it is the specific coordination work between services that seems to be central. The authors highlight the importance of implementing national and regional guidelines to guide this transition, that could be useful and effective without requiring additional staffing.

Reis et al. aimed to demonstrate the effectiveness of a behavioral prevention program on sexual abuse for children with intellectual disabilities. While girls in the intervention group (*n* = 64) showed improvements in prevention knowledge compared to the control group (*n* = 39), they did less so in prevention behavior, and field tests with realistic seduction situations showed no improvement. This last point is important because, as the authors emphasize, naturalistic contexts are essential to provide evidence of the effectiveness of preventive interventions for children with intellectual disabilities.

Finally, Lincke et al. studied attitudes toward innovative therapeutic approaches (e-mental health and home treatment) in the German health care system. Less than one in five participants preferred online treatment approaches, while more than half could imagine being treated at home. Younger subjects were more open to online therapy approaches, while people with a low level of education often preferred traditional treatment. Interestingly, the level of acceptance of online therapy did not increase significantly during the first months of the COVID-19 pandemic. The results of this study therefore highlight the need to inform about and promote these innovative treatments, whose diffusion and level of acceptance still seem moderate.

In sum, this Research Topic provides new evidence on: (i) acupuncture treatment of tic disorder in children; (ii) factors that can ensure a quality transition in psychiatry; (iii) an innovative behavioral prevention program on sexual abuse for children with intellectual disabilities; (iv) attitudes of the general population toward innovative therapeutic approaches.

In conclusion, we believe it is important to encourage local and global networks of cooperation and collaboration and the aim of this Research Topic, coordinated by two clinician-scientists, one American and one French, is to contribute—in a modest way—to the advocacy on the ground of these crucial issues, in order to encourage stakeholders to demand socially justifiable and equitable resources. As we have stated several times before (9, 10), promoting child and adolescent mental health is imperative to reduce the burden of mental health problems in future generations and to enable the successful and satisfying development of vulnerable children and adolescents (11).

## Author contributions

AR and CK are the two topic editors of this Research Topic and take responsibility for the views expressed in this editorial. AR wrote the first version of this editorial. CK extensively reworked the editorial. Both authors contributed to the article and approved the submitted version.

## Conflict of interest

The authors declare that the research was conducted in the absence of any commercial or financial relationships that could be construed as a potential conflict of interest.

## Publisher's note

All claims expressed in this article are solely those of the authors and do not necessarily represent those of their affiliated organizations, or those of the publisher, the editors and the reviewers. Any product that may be evaluated in this article, or claim that may be made by its manufacturer, is not guaranteed or endorsed by the publisher.
